# Influence of the exposed anatomic sites on the human in vivo percutaneous absorption of the amphiphilic 2-phenoxyethanol

**DOI:** 10.1007/s00204-025-04212-y

**Published:** 2025-10-29

**Authors:** Julia Hiller, Elisabeth Eckert, Thomas Jäger, Michael Bader, Andrea Kaifie, Thomas Göen

**Affiliations:** 1 Institute and Outpatient Clinic of Occupational, Social and Environmental Medicine, Friedrich-Alexander-Universität Erlangen-Nürnberg, Erlangen, Germany; 2 Bavarian Health and Food Safety Authority, Erlangen, Germany; 3 BASF SE, Corporate Health Management, Ludwigshafen, Germany

**Keywords:** Regional variation, Skin penetration, 2-Phenoxyethanol, Biomonitoring, Percutaneous absorption

## Abstract

Percutaneous penetration is an important human exposure route, but it is influenced by multiple factors and can vary greatly between different substances and exposure conditions. Furthermore, skin characteristics such as thickness of different anatomic sites may have an effect on percutaneous absorption. Despite a potential relevance for safety measures, little is yet known for occupationally relevant skin sites, like hands. We conducted a human in vivo study aiming to identify the effect of anatomic sites on percutaneous absorption of 2-phenoxyethanol. Two human volunteers were dermally exposed to 2-phenoxyethanol (loading dose: 0.2 mg/cm^2^ skin) at seven different anatomic skin sites with areas of 200 and 400 cm^2^, respectively. Blood and urine samples were collected and analyzed for 2-phenoxyethanol and its two main metabolites up to 48 h post-exposure. Relative absorption ratios showed moderate site differences ranging from 0.89 to 1.64 (compared to the forearm) as the overall renal recovery was between 23 and 43% of applied 2-phenoxyethanol. Inter-individual variability was in a similar range. A notable influence of the exposed site could, however, be observed for the penetration speed which was highest at the forehead and neck, and slowest at the hand palms. Differing ratios of unmetabolized 2-phenoxyethanol and its main metabolite found in blood point to a site-depending intradermal metabolism. This study revealed major findings concerning the velocity of percutaneous penetration at different skin sites, which is relevant for adequate skin protection. The obtained data broaden the knowledge about site-related variations in percutaneous absorption and thus can improve future toxicological evaluations.

**Supplementary Information:**

The online version contains supplementary material available at 10.1007/s00204-025-04212-y.

## Introduction

Percutaneous absorption is an important human exposure route for various substances. However, its mechanisms are complex and multiple factors can influence the amount and rate of percutaneous absorption. In particular, the physicochemical properties of the substance (such as molecular mass, lipophilicity, etc.) and skin characteristics (such as thickness, hydration, sebum content, skin appendages, blood flow, transepidermal water loss (TEWL)) are of high importance (Law et al. 2020). Additionally, vehicle or substance formulation and exposure conditions (dose, duration, surface area, occlusion, temperature, humidity) can influence the penetration behavior (Law et al. 2020; Kilo et al. 2020).

With various skin inherent factors known to affect penetration, it can be assumed that percutaneous absorption will also be affected by the characteristics of exposed skin sites, as human skin can exhibit different conditions depending on the associated anatomical site (Nedelec et al. 2016; Tagami 2008). Accordingly, a high site-related variability has already been described for some substances, such as hydrocortisone and pesticides (Feldmann and Maibach 1967; Maibach et al. 1971). Law et al. (2020) recapped that absorption rates tend to be generally faster in hair follicle-rich sites and slower at sites where a thicker stratum corneum can be found. A recent review (Bormann and Maibach 2020) updated earlier reviews and evaluated the available literature on the effect of anatomical site on human percutaneous penetration including medication patch data. The authors described several human in vivo studies investigating regional variation for percutaneous penetration. Human biomonitoring data from urinary excretion were available for hydrocortisone, testosterone, benzoic acid, benzoic acid sodium salt, acetylsalicylic acid, caffeine, malathion, parathion, carbaryl, and pyrene tested in 14 different settings by eight studies (Feldmann and Maibach 1967; Maibach et al. 1971; Britz et al. 1980; Rougier et al. 1986, Rougier et al. 1988; Lotte et al. 1987; VanRooij et al. 1993; Oriba et al. 1996). Four of those studies, however, tested only two sites, with three of them comparing only the forearm and genitalia.

With respect to practical implications of varying percutaneous absorption depending on the exposed skin site, some sites are of special interest, either in the medical field with transdermal drug delivery systems (TDDS) or at the workplace. Especially in occupational settings, body parts such as the face, neck, and hands are particularly important since they can be particularly exposed. However, human in vivo studies including the hands are rare (Feldmann and Maibach 1967; Maibach et al. 1971; Van Rooij et al. 1993).

Many of the substances evaluated so far for site-specific dermal penetration rates are primarily lipophilic. However, amphiphilic substances, such as N-methylpyrrolidone and 2-phenoxyethanol (PhE), can also readily penetrate the skin (Bader et al. 2005; Eckert et al. 2025).

2-Phenoxyethanol is an organic compound used as a preservative in the medical field (disinfectant or vaccine solutions), in hygiene, and cosmetic products (body lotion, shampoo, baby wipes) and at the workplace, for example as a biocide in metal working fluids (ECHA 2025; Hartwig et al. 2019). In both applications, the main route of exposure is likely to be dermal. Due to its pronounced percutaneous penetration behavior, low toxicity in man (derived no effect level (DNEL) for long-term dermal exposure by ECHA: 10.42 mg/kg bw (ECHA 2025)), and available suitable biomonitoring methods (Jäger et al. 2022, 2024; Eckert et al. 2024, 2025), PhE has emerged as an ideal substance for evaluating percutaneous absorption in small-scale exposure scenarios to broaden the knowledge about site-specific variation of penetration.

This research, therefore, aims to investigate the effect of the anatomic skin site on the percutaneous absorption of PhE in humans by the assessment of PhE and its main metabolites in blood and urine. The volunteer study focused on sites particularly relevant for occupational settings and also of consumer applications.

## Materials and methods

### Study population and study design

Two volunteers (one male; one female; details in table S1 online resource 3) were exposed dermally to PhE at different skin sites in a series of individual experiments. The volunteers were healthy adults, who were not occupationally exposed to PhE and who were instructed to avoid potentially PhE-containing consumer products (e.g., skin creams, lotions) for at least 3 days prior to the experiment and up until the end of the follow-up sampling period. The seven PhE-exposed anatomic sites included: neck and forehead, back, abdomen, thigh, forearm, both palms of the hands, and both dorsal hands (Fig. S1 online resource 2). The male volunteer did not have a beard, but did not shave during exposure to avoid irritating effects. At least 3-week breaks were maintained between the individual experiments to ensure complete elimination of experimental PhE exposure from the body.

The study was approved by the local ethics committee of the Friedrich-Alexander-University Erlangen-Nürnberg, Germany (No. 296_19B). Prior to inclusion, all participants gave their written informed consent after information about the aims and risks of the study.

Each exposure experiment was carried out with a targeted single dermal loading dose of 0.2 mg/cm^2^ PhE. The dimension of each exposure site was set to 400 cm^2^, except for the two hand exposure scenarios, where 200 cm^2^ each was exposed. The topical application was performed using non-occlusive conditions with an ointment containing 10.0% PhE in a standard pharmacy cream formulation (“DAC Basiscreme”). The absolute applied doses in each experiment were determined by weighing out the empty ointment containers. Doses were 87.5 ± 3.7 mg PhE and 44.0 ± 1.0 mg PhE for the 400 cm^2^ and 200 cm^2^ skin areas, respectively. After administration, the application sites were left uncovered for at least 6 h to avoid unintended losses by contact with clothing and to enable a complete absorption of the ointment. More details for the rationale of the chosen exposure scenarios are given in online resource 1.

To evaluate the percutaneous absorption of PhE, the two main metabolites phenoxyacetic acid (PhAA) and 4-hydroxyphenoxyacetic acid (4-OH-PhAA), which have been shown to account for more than 99% of the renally excreted PhE dose in earlier toxicokinetic studies (Eckert et al. 2024, 2025), were determined in blood and urine samples. Additionally, unmetabolized PhE in blood was analyzed, as relevant amounts were found in a metabolism study following dermal PhE exposure of the abdomen in five volunteers (Eckert et al. 2025).

Sample collection took place via complete fractioned urine sampling for 48 h and drawing of blood samples (using EDTA monovettes) at 1, 2, 3, 4, 5, 6, 9, 12, 15, 24, 33, and 48 h post-exposure. Additionally, pre-exposure samples of blood and urine were collected immediately prior to exposure (which was scheduled early in the morning) to monitor background levels for each experiment. The volunteers were asked to provide a total void urine sample every time a blood sampling was scheduled. If further urination was necessary in between, those samples were collected and the times recorded.

For accurate determination of the individual urine volumes, the collected samples were weighed and then stored frozen at −20 °C until analysis. Blood samples were also stored frozen at −20 °C until analysis.

### Chemical blood and urine analysis and materials

The PhE metabolites PhAA and 4-OH-PhAA in urine and blood were determined using LC–MS/MS analysis (without an additional hydrolysis step due to unconjugated metabolite presence) as described in a previously published procedure (Jäger et al. 2022). Details on sample preparation, used reagents and chemicals, instrumentation, and applied procedures have been published there. Similarly, the details of the methodical procedure applied for determination of PhE in blood have also been described elsewhere (Jäger et al. 2024). Briefly, unmetabolized PhE was determined using GC–PCI-MS/MS analysis, including a hydrolysis step, as PhE was shown to be excreted in significant amounts in the form of conjugates. The limits of quantification (LOQ) were 10 and 20 µg/L for PhAA and 4-OH-PhAA in urine, respectively, as well as 6, 10, and 2 µg/L for PhAA, 4-OH-PhAA and PhE in blood, respectively.

### Data processing and statistical analysis

The two main metabolites, PhAA and 4-OH-PhAA, account for almost all excreted PhE doses in urine; however, the relative shares of PhAA to 4-OH-PhAA can vary significantly on an inter-individual basis (Eckert et al. 2024, 2025). Therefore, the sum of both metabolites in urine was used to compare the percutaneous absorption rates depending on the exposed skin site. The metabolite sum was then put in relation to the respective application dose giving urinary excretion factors (F_UE,i_) expressed as PhE dose equivalents (in %). The following calculation steps were performed: The absolute excreted amounts [mg] of PhAA and 4-OH-PhAA in the respective sampling periods were calculated using the analyte concentration [mg/L] and volume [L] of the urine sample ($Ci\times Vi)$ and thereafter converted to µmol (molecular weight of analytes: PhAA 152.15 g/mol; 4-OH-PhAA 168.15 g/mol). The sum of both amounts in µmol was related to the administered PhE dose (in µmol) giving the F_UE,i_ of the respective sampling period. Subsequently, renal excretion rates (R_E_, in %/h) were calculated with the following equation using the respective *F*_*UE,I*_, the sampling time *t*_*i*_ after dermal exposure (in h), and the sampling time of the previous sample *t*_*i-1*_ (in h):

$${R}_{E,i}= \frac{{F}_{UE,i}}{{t}_{i}- {t}_{i-1}}.$$

If more than one urine sample was collected by the volunteers in the time period between the blood samples, the amounts of analytes (in µmol) of each urine sample in that period were first calculated and summed up to achieve the total amount of excreted analytes for this sampling period. Therefore, several urine samples were considered together to obtain the same sample periods as for blood (1, 2, 3, 4, 5, 6, 9, 12, 15, 24, 33 and 48 h post-exposure).

In blood, the measured concentrations in mg/L were used to evaluate the absorption of PhE and its elimination kinetics.

Data evaluation and statistical analyses were done with Microsoft® Excel® 2021 and Origin® 2024b using a common approach to evaluate the toxicokinetic characteristics as described in earlier studies (Fischer et al. 2025; Eckert et al. 2024, 2025). Samples below LOQ were replaced by LOQ/2 to be factored in the kinetic calculations.

For comparison of the total renal recovery (= TRR) of PhE, the F_UE,i_ of each sample was summed up cumulatively and plotted against time post-exposure. Logistic growth curves for each exposure scenario were fitted (using the combined volunteer data). Differences in the temporal course of the cumulative recovery were evaluated by setting the overall TRR after 48 h for each location site as 100% (= site-specific maximum) and calculating the relative share (in %) of the overall 48 h TRR at certain timepoints with the following formula:

$${RS}_{i}=\frac{{TRR}_{i}}{{TRR}_{48h}}.$$

The t_rec50_ (= time to 50% recovery of the percutaneously penetrated dose) was retrieved for each logistic growth curve. As performed in earlier studies (Feldmann and Maibach 1967; Maibach et al. 1971), the forearm was used as the reference location to evaluate differences in overall absorption rates among studied sites.

Additionally, temporal progression data were also obtained by plotting blood levels against the exact sampling time points, respectively, in urine by plotting the renal excretion rates *F*_*UE,I*_, against the midpoint of the respective sampling periods (*t*_*i,m*_ = *t*_*i-1*_ + (*t*_*i*_ – *t*_*i-1*_)/2). Log-normal curves were fitted and the respective peak levels (C_max_/R_E,max_) and t_max_ (= time post-exposure at C_max_/R_E,max_) subtracted from the curves.

To calculate terminal half-lives (t_1/2_), the temporal progression data were ln-transformed and a linear regression was fitted to the segment of constant decline (assuming a first-order elimination process) to obtain the slope (k_el_, elimination rate constant). Half-lives were then calculated using the formula:

$${t}_{1/2}=\frac{\mathrm{ln}(2)}{\left|{k}_{el}\right|}.$$

To ensure a comparable approach, the same time segments (starting after the latest observed curve maxima) were used for each “analyte–matrix pair” regardless of the location site or volunteer.

## Results

Post-exposure, both metabolites could be reliably detected in urine with detection rates of 100% for PhAA and 97% for 4-OH-PhAA among all samples. The detection rate in blood was lower: PhAA was above LOQ in 84% of the samples, PhE in 57%, and 4-OH-PhAA only in 5%. Therefore, 4-OH-PhAA in blood was not used for any further evaluations. For some blood samples, a PhE analysis could not be performed due to insufficient amount of sample material. In total, 9 out of 182 samples were affected. With one exception (at 15 h), all of those came from the last two post-exposure samples (33/48 h).

Overall, pre-exposure analyte levels in blood and urine were mostly in the range or below the levels at the end of the follow-up after 48 h.

### Effect on the absorption rate

A comparison of the overall percutaneous absorption was performed by evaluating the cumulative PhE recovery in urine, where the female volunteer P2 generally showed a higher recovery rate than the male volunteer P1 in six out of seven sites (as seen in Fig. S2 online resource 2, only exemption “thigh”). TRR after 48 h ranged from 22.8% to 39.4% in volunteer P1 (mean: 30.9 ± 6.0%) and 19.4% to 57.7% in volunteer P2 (mean: 39.0 ± 11.4%) for the different exposure sites. The highest inter-individual difference was observed for the dorsal hands (Fig. S2 online resource 2).

**Fig. 1:**
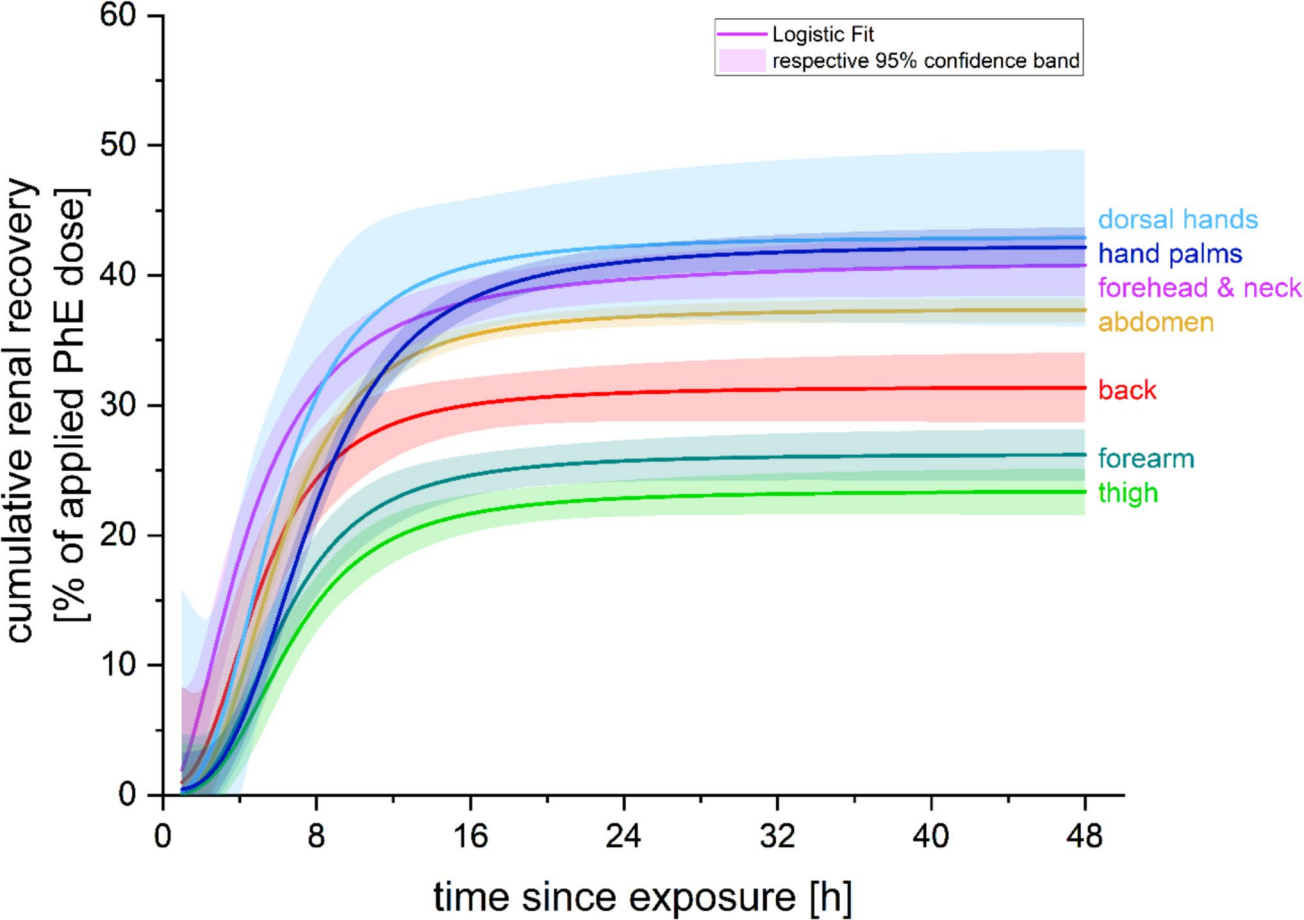
Course of cumulative renal recovery of applied dermal PhE dose (in %) over time per exposure site (per logistic growth fit for combined subject data)

The logistic fits for the combined volunteer data gave a similar range of variance between sites as seen on an individual basis (Fig. 1, Tab. 1). Overlapping ranges of the fits’ 95%-confidence bands for the four locations with the highest urinary recovery rates (dorsal hands, hand palms, forehead and neck, abdomen) and among the two locations with the lowest TRR (forearm, thigh) demonstrate only moderate differences in the overall absorption rate of PhE with respect to the exposed skin sites. Relative absorption ratios in relation to the forearm ranged from 0.89 to 1.64 (Tab. 1).

**Table 1:** Total renal recovery (TRR), relative absorption ratio (in relation to the forearm), and t_max_ of elimination kinetics in the blood and urine (from combined fit, and range of individuals) per anatomic site following dermal PhE administration to two volunteers on seven different anatomic sites

	TRR (based on logistic data fit (95% CI))	FUE_skin site_/FUE_forearm_	t_max_ [h]		
			PhE in blood	PhAA in blood	∑PhAA + 4OH−PhAA in urine
Dorsal hands	42.9% (36.1–49.7)	1.64	2.1 (2.1–2.6)	3.4 (3.4–4.0)	4.5 (4.4–4.9)
Hand palms	42.2% (40.7–43.7)	1.61	2.9 (1.8–3.7)	5.1 (4.9–5.8)	5.4 (5.2–5.4)
Neck and forehead	40.8% (38.4–43.2)	1.56	0.8 (0.7–0.9)	2.1 (2.1–2.1)	2.0 (1.9–2.2)
Abdomen	37.4% (36.4–38.3)	1.43	1.7 (1.1–2.2)	3.9 (3.3–4.6)	4.5 (3.9–5.2)
Back	31.4% (28.7–34.1)	1.20	1.3 (0.5–1.2)	2.9 (2.5–4.2)	3.2 (2.5–4.2)
Forearm	26.2% (24.2–28.2)	1.0	1.9 (1.8–2.1)	3.8 (3.0–5.8)	4.5 (4.1–5.8)
Thigh	23.4% (21.6–25.1)	0.89	1.7 (1.6–1.7)	4.4 (4.4–4.4)	5.1 (4.9–5.3)

### Time effects and elimination kinetics

The courses of cumulative renal recovery over time (Fig. 1) with their respective turning points and begin of saturation phases showed varying penetration speeds depending on the exposed skin sites. Additionally, the relative shares of TRR at certain time points differed between the sites (Tab. S2 online resource 3). The relative recovered shares for forehead and neck and to a lesser extent for the back were higher in the first 6–8 h, while especially the palms showed comparatively low renal recovery rates during this period. However, after 16 h post-exposure more than 90% of the overall recovery was completed for all sites (Table S2 online resource 3). All site-specific recovery time courses had aligned after 16–24 h and showed very comparable relative shares. Accordingly, t_rec50_ ranged from 4.5 to 7.7 h (mean: 6.0 ± 1.1 h) (Table S2 in online resource 3).

**Fig. 2:**
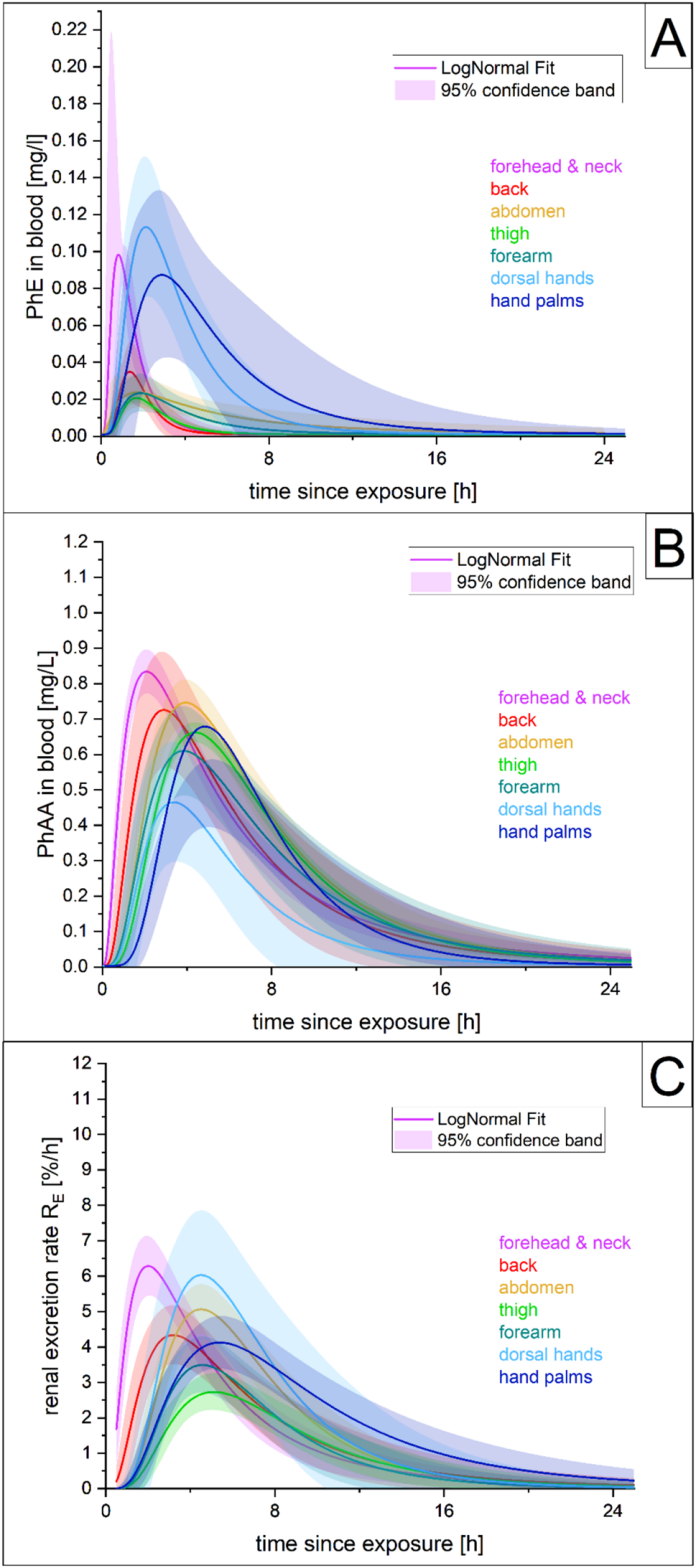
Log-normal fits for the site-specific elimination kinetics of PhE (A) and its metabolite PhAA (B) in blood and the sum of the two main metabolites (FUEsum) in urine (C), after dermal exposure with phenoxyethanol

Figure 2 shows the site-specific blood concentration kinetics of PhE and its metabolite PhAA (A & B) and the kinetics of the renal excretion rate (R_E_) for the sum of the metabolites in urine (C) after the dermal exposure with PhE for the combined volunteer data. As in the cumulative recovery, some inter-individual differences, especially with respect to the peak blood levels or the highest R_E_, can be observed (Fig. S3-S5 online resource 2). In urine (Fig. S5 online resource 2), the female volunteer P2 again showed generally higher excretion rates than the male P1 in six out of seven sites. For PhE and PhAA in blood (Fig. S3 & S4 online resource 2), the female volunteer P2 again tended to show higher concentration levels; however, this effect was less pronounced than in urine, as only about half of the sites showed higher variations in the absolute peak levels.

In general, PhE blood levels showed a higher variance depending on the exposed skin site than the metabolites. Especially, the site forehead and neck and the two hand scenarios showed higher peak blood concentrations than the other four locations, despite the fact that the blood concentrations were not corrected for the lower total exposure dose (50%) in the hand scenarios. However, the blood levels of the metabolite PhAA were still significantly higher than for unmetabolized PhE (peak concentrations higher by a factor of 4- to 8-fold for forehead and neck, dorsal hands, and hand palms; and 21- to 31-fold for all other locations). In contrast, the elimination curve variance between the sites was rather small for the metabolites, and even more for PhAA in blood than R_E_ in urine (Fig. 2).

Figure 2 also clearly illustrates that in the case of the forehead and neck scenario, blood levels and R_E_ consistently peaked earlier, and shorter t_max_ times were observed (Table 1), indicating an earlier onset and a faster penetration rate for this skin site. The second fastest t_max_ was seen for the back, and the latest for the palms; all others were within a similar range (Table 1).

Peak levels and half-life data are reported in online resources 2 and 3 (Tables S3, S4). As also visible from the elimination curves (Fig. 2), forehead and neck again tended to be among the sites with the highest peaks of blood levels or R_E_ with all other sites varying considerable depending on the analyte and matrix; therefore, no particular consistent order of sites could be otherwise established.

Due to an insufficient number of data points (levels above LOQ), the elimination half-life calculation could not be performed for PhE in blood. For R_E,_ the time frame 5.5–40.5 h post-exposure was used and showed a very tight range of 4.4–4.7 h. For PhAA in blood, the segment 6–24 h was used (as levels often dropped below LOQ around 24 h). Here, t_1/2_ showed a little higher variance and ranged between 2.8 and 4.4 h; however, only t_1/2_ of the thigh was notably longer than the overall trend.

## Discussion

The present study investigates the influence of the exposed anatomic site on percutaneous absorption of the amphiphilic substance PhE. While the effect on the renal recovery of PhE metabolites was rather moderate and stayed within the inter-individual variability, a pronounced influence of the exposed skin sites could be observed regarding the penetration speed of PhE. The fastest penetration was observed for the forehead and neck, whereas PhE penetrated most slowly through the palms of the hands. With both the unmetabolized PhE and its main metabolites analyzed in this study, intradermal metabolism could also be included in the appraisal of site-specific percutaneous absorption for the first time.

To date, only a limited number of human in vivo studies have investigated the impact of different anatomic skin sites on percutaneous absorption and have shown rather differing results as reported by a recent summary by Bormann amd Maibach (2020). In general, these studies observed variation in the penetration rate between different sites, mostly in a range of about 0.5- to 4-fold compared to a reference site (forearm or upper outer arm). However, in some cases, even higher factors were described. Those high-scale variations mostly affected sites with special properties, such as genitalia or axilla, where possibly an occlusion effect may have supported the absorption additionally. The by far highest penetration rate has been reported for hydrocortisone application on the scrotum (42-fold above the forearm) (Feldmann and Maibach 1967). A compilation of urine data from literature, focusing on locations without special properties or potentially occlusive conditions, of regional variation in percutaneous absorption, as compiled by Bormann and Maibach (2020), is included in online resource 3 (Table S5). In summary, a higher absorption was often described for the head, neck, and genitalia compared to the trunk, back, and thighs that exhibited lower absorption rates (Bormann and Maibach 2020).

In this study, the observed variation of total renal recovery of PhE was rather small and ranged from 0.89- to 1.64-fold in reference to the forearm. This is in accordance with some of the existing data that observed lower variation up to a range of twofold for pyrene (Van Rooij et al. 1993), caffeine, and acetylsalicylic acid (Lotte et al. 1987), as well. Similarly, all data derived from plasma pharmacokinetics for medical patches or TDDS with pharmaceutics showed only minimal variations depending on the exposed site (when excluding the buccal mucosa) between 0.7- and 1.2-fold with respect to the area under the curve (AUC) (Bormann and Maibach 2020). For comparison of the total substance absorption, however, renal recovery data are considered to be the more robust data for substances with relevant renal elimination, while blood concentrations are only a reflection of the current circulating levels without knowing the total substance amount present in blood. The AUC is therefore only a mathematical estimate for a total sum.

In our study, percutaneous absorption of PhE occurred faster over the skin of the forehead and neck and the slowest through the palms as demonstrated by the t_max_ of all analytes in blood and urine and differing relative shares of TRR at earlier sampling time points. The back was found to be the second fastest location for absorption. Interestingly, penetration velocity did not seem to influence the overall absorbed amount, as the palms with a slow penetration also had a high TRR.

Time effects have also been discussed in literature. Somewhat varying excretion kinetics have been observed for hydrocortisone, pesticides, and pyrene (Feldmann and Maibach 1967; Maibach et al. 1971; VanRooij et al. 1993). The temporal breakdown was rather vague (≥ 12-h intervals) for hydrocortisone and did not allow a closer insight into the critical early penetration phase. However, the maximum excretion rate for the plantar foot was reported to occur at the the latest (days 3 and 4) (Feldmann and Maibach 1967). A finer differentiation was reported for the investigated three pesticides, but not all were tested on all skin sites (Maibach et al. 1971). For carbaryl as an almost complete penetratant (about 70% recovery for the forearm and jaw angle), higher absorption rates were described in the earlier time periods (4-h intervals in the first 12 h) for the jaw angle. For malathion with a much lower overall absorption, the data showed a higher relative share of the total recovery within the first 8 h for the axilla (51%) and a relatively reduced share for the foot and palm (9 and 13%, respectively). The forehead was the site with the second highest relative share (37%) in the first 8 h. In contrast, the variation among the relative shares in the first 8 h for parathion was mostly lower (between 3 and 13%) with only the scrotum as a faster outlier (21%). VanRooji et al. (1993) reported significantly differing times in which half of the total 1-hydroxypyrene was excreted (from 8.2 to 18.9 h) with the neck as the fastest location and the hand as the slowest. In summary, our observations are therefore also supported by trends seen in the literature with a faster penetration over the face and a delayed absorption for the palms or foot (Maibach et al. 1971; VanRooij et al. 1993).

These site-specific time effects might be explained by different skin characteristics and inherent PhE properties. Firstly, skin thickness differs throughout the body, and a thinner stratum corneum and skin likely leads to a faster (and potentially greater) penetration. A study by Nedelec et al. (2016), however, reported only small differences in skin thickness between the face parts and the volar hand, but the highest thickness for the back. On the other hand, a review by Tagami (2008) concluded that the stratum corneum of the facial skin is thinner and functions differently compared to other body parts with the exception of the neck. Due to these differences, the facial skin shows a poorer barrier function and faster turnover. Law et al. (2020) also described that the absorption rates are slower where the stratum corneum is thicker.

Secondly, the blood perfusion of skin varies among the different sites, as well. Marrakchi and Maibach (2007) reported that various sites around the face and neck showed a higher baseline blood perfusion compared to the forearm. Furthermore, skin appendages such as hair follicles and sweat and sebaceous glands are disseminated differently across different body parts and can influence percutaneous absorption (Law et al. 2020). For example, a faster penetration is generally described especially for hair follicle-rich areas (Law et al. 2020). Facial skin is also reported to be particularly rich in sebaceous glands, which can lead to higher skin sebum content (Tagami 2008; Firooz et al. 2012). However, the effect of sebum on the percutaneous absorption of xenobiotics is unclear (Schneider et al. 2016). An effect of hair follicle density may be especially relevant with regard to the palms and the slower penetration seen there, as glabrous skin only exists on the palms and soles and is characterized by its missing hair follicles and its higher durability against mechanical forces. Lastly, substance characteristics are likely to play a role, as well. Temporal differences among sites are possibly more pronounced or more easily detected for substances which show a higher overall absorption.

PhE has shown a relatively high percutaneous absorption rate of about 45% at the abdomen (Eckert et al. 2025), which reduces the possible rate of increase when more permeable regions are exposed. Furthermore, its molecular size is much smaller than that of hydrocortisone, for which the highest variations were described (Feldmann and Maibach 1967), and more similar to some of the other substances (like benzoic acid or pyrene) with also mostly moderate variations (Lotte et al. 1987; VanRooij et al. 1993). Often, smaller molecules are able to penetrate the skin more easily (Law et al. 2020). Accordingly, the physico-chemical properties of PhE may be an argument for the only moderate variations observed for the overall absorption rate. Skin thickness might also explain the inter-individual differences in TRR, where volunteer P2 (female) generally showed a higher recovery than volunteer P1 (male) as Nedelec et al. (2016) also reported that men generally had a thicker skin than women.

Other sex-specific effects might also be discussed in the light of the inter-individual differences seen in our data. Besides skin thickness, sex differences have also been described for other skin properties which might influence percutaneous absorption, such as sebum production, skin pH, and hydration or water loss (Luebberding et al. 2013; Man et al. 2009; Zhao et al. 2021). A higher sebum production, as often described for males (Luebberding et al. 2013; Man et al. 2009; Zhao et al. 2021), might reduce the overall penetration, especially for an amphiphilic substance like PhE. A lower water loss and higher skin hydration have also been described in men (Luebberding et al. 2013, Man et al. 2009), though not in all studies (Zhao et al. 2021). Skin pH, however, tends to be consistently lower in males (Luebberding et al. 2013; Man et al. 2009; Zhao et al. 2021) and might also affect percutaneous absorption. Law et al. (2020) pointed out that a basic pH environment better promotes deionized forms of chemicals which in turn generally leads to better absorption through the skin. Therefore, a higher skin pH in females might also facilitate a higher penetration rate. Hair follicle density, however, does not seem to affect potential sex-specific effects, as it did not differ between males and females (Seago and Ebling 1985). All in all, due to the chosen study design, a reliable sex-specific evaluation is neither possible nor scientifically justified. Furthermore, considerable inter-individual differences in the overall recovery of PhE following dermal exposure in five volunteers were also found in the dermal toxicokinetic study by Eckert et al. (2025). Thus, a reliable assessment of sex-specific differences would require a significantly higher number of subjects.

In the study by Eckert et al. 2025, the dermal exposure was carried out with a single dose of 0.4 mg PhE/kg bw on 800 cm^2^ abdomen skin which corresponded to absolute doses between 23 and 36 mg PhE, giving a dermal loading dose in the range of 0.03–0.04 mg/cm^2^. In comparison, in this study, a fixed dermal loading dose of 0.2 mg/cm^2^ was used (higher by a factor of 5–7). Due to the reduced exposure areas, this resulted in singles doses between 1.1 and 1.4 mg PhE/kg bw (400 cm^2^) and 0.5–0.7 mg/kg bw (200 cm^2^). Therefore, the total exposure dose here was only higher by a factor of 1.5–3. Eckert et al. 2025 found an overall percutaneous absorption rate for the abdomen of 44.9%, which is in a similar range as seen with the combined volunteer data for the abdomen in this study (37.4%). Comparable results are also found for the temporal toxicokinetic data (t_max_; t_1/2_) of PhE and its metabolites in blood and urine at the abdominal site. The unmetabolized PhE/metabolite PhAA ratio at c_max_ level in the blood of 31-fold for the abdominal exposure here was also very similar to the about 30-fold higher levels described by Eckert et al. 2025.

The elimination kinetics for unmetabolized PhE in blood showed the highest variance among the investigated sites. In particular for the forehead and neck and the two different hand sites (palmar and dorsal), higher peak concentrations of PhE in blood were observed compared to the other four locations, considering the reduced exposure dose at the hands. Especially, the ratio between the maximum blood concentrations of PhE and PhAA varied greatly depending on the exposed skin site and was only about four- to eightfold for the forehead and neck and both hand sites. For all other locations, the 21- to 31-fold higher PhAA levels were in accordance with what Eckert et al. (2025) found. As PhAA maximum levels in blood were more than 400-fold higher than PhE after oral exposure indicating a considerable first-pass effect (Eckert et al. 2024, 2025), these site-specific ratios cannot be explained by a potential oral co-exposure by (hand–)mouth contamination. There are various possible explanations: for the forehead and neck, the especially fast penetration and the early peak might lead to a higher PhE peak level in blood compared to the metabolized PhAA level. If conversion from PhE to PhAA in the liver after systemic absorption is at a constant rate and this rate is slower than the initial PhE absorption, the observed PhE levels in blood might be higher. This explanation, however, cannot be transferred to the hands, as they showed a delayed penetration compared to the forehead and neck. Here, a partly intradermal metabolization and regional variation in intradermal enzymes might explain the relatively higher PhE levels in blood for the hands. Biotransformation of PhE to PhAA is carried out mostly in the liver, catalyzed by cytosolic alcohol dehydrogenase (ADH) and aldehyde dehydrogenase (ALDH). However, in vitro incubation of PhE with rat skin also resulted in small amounts of PhAA formation (about 5% capacity of the liver enzyme activity (Roper et al. 1997; SCCS 2016). Tagami (2008) reported a more metabolically active skin of the face. Therefore, the metabolic activity in the skin of the hands would be reduced compared to other regions. This might also explain higher PhE amounts in blood.

Data on this topic only rarely exists and none specifically studying the palms. Cheung et al (1999) reports the detection of several classes of ADH and ALDH in human skin samples (from foreskin, breast and abdomen). Densitometric analysis showed that the levels of enzyme expression in the different skin locations varied: class I and II ADH expression was significantly reduced for the foreskin compared to the breast and abdomen, class II ADH showed no differences, while class 1 and 3 ALDH expression was higher in foreskin. Furthermore, Calman et al. (1970) report more active steroid dehydrogenases in skin areas with dense sebaceous glands and therefore a regional variation in intradermal enzyme activity in humans. For hamster, skin variations in intradermal serotonin metabolism were found with much higher activity in ear skin compared to corpus skin (Slominski et al. 2002). Similarly, a regionally varying distribution of glutamine synthetase levels in human and rat skin was reported by Danielyan et al. (2009).

Lastly, the strength and limitations of the study need to be addressed. One study limitation is the low number of volunteers. However, both sexes and a certain age range are present, which on the other hand may also partly explain the observed inter-individual differences. Human in vivo studies on percutaneous absorption are complex and require a lot of time and resources as well as demand a high compliance of the participants with the protocol which naturally limits the accessibility of appropriate volunteers.

A clear strength of this study is the comprehensive exposure assessment including blood and urine as well as the monitoring of the main metabolites and the parent compound. This not only allows a more complete evaluation of the actual percutaneous absorption, but can also be used to look into more specific metabolism effects. Moreover, the assessment did not focus on the total absorption exclusively, but also the effect on the penetration kinetics.

## Conclusion

The present study illustrates the influence of the anatomic skin site on the percutaneous absorption of 2-phenoxyethanol. Seven sites were selected, some of which are typically exposed in occupational settings, where a site-specific influence on percutaneous absorption is of particularly high importance for potential safety measures. The hands (dorsal and palms) were included in an evaluation of regional variation for an amphiphilic substance, for the first time in a human in vivo study.

A notable site-specific effect on the penetration velocity could be observed. Penetration of the amphiphile PhE via the forehead and neck occurred faster than through other body parts and was delayed for the palms. Accordingly, t_rec50_ ranged from 4.5 to 7.7 h. Only a moderate site-specific variation was observed for the overall absorption. Higher variations reported in other studies can probably be ascribed to other substance properties. In this study, more pronounced site-specific effects might have also been concealed by other influence factors, such as inter-individual variation or sex. Moreover, the results suggested a site-depending intradermal metabolism by the assessment of the parent compound and its main metabolites. However, this finding needs confirmation by further investigations.

The presented results broaden the knowledge on site-related variation in percutaneous absorption, in particular for less studied sites like the palms and amphiphilic substances, and may therefore be useful for future toxicological evaluations of comparable compounds.

## Supplementary Information

Below is the link to the electronic supplementary material.

Supplementary file1 (PDF 22 KB)

Supplementary file2 (PDF 392 KB)

Supplementary file3 (PDF 204 KB)

## Data Availability

The datasets generated in this study are available from the corresponding author upon reasonable request.

## Abbreviations

bw: Body weight
DNEL: Derived no effect level
F_UE_: Urinary excretion factor
LOQ: Limit of quantification
NOAEL: No observed adverse effect level
4-OH-PhAA: 4-Hydroxyphenoxyacetic acid
PhAA: Phenoxyacetic acid
PhE: Phenoxyethanol
R_E_: Renal excretion rate
t_1/2_: Half-life
