# Supplementary material for: Influence of the exposed anatomic sites on the human in vivo percutaneous absorption of the amphiphilic 2-phenoxyethanol
Source: Arch Toxicol. 2025 Oct 29;100(2):557–67. doi: 10.1007/s00204-025-04212-y (PMC12886243; doi:10.1007/s00204-025-04212-y)
Supplement: Supplementary file 1 — Supplementary file1 (PDF 22 KB) [file 204_2025_4212_MOESM1_ESM.pdf]

Online Resource 1

Title: Influence of the exposed anatomic sites on the human in-vivo percutaneous absorption of the amphiphilic 2-phenoxyethanol

In: Archives of Toxicology

By: Julia Hiller[1]*, Elisabeth Eckert[1,2], Thomas Jäger[3], Michael Bader[3], Andrea Kaifie[1], Thomas Göen[1]

[1] Institute and Outpatient Clinic of Occupational, Social and Environmental Medicine, Friedrich-Alexander-Universität Erlangen-Nürnberg, Erlangen, Germany

[2] Bavarian Health and Food Safety Authority, Erlangen, Germany

[3] BASF SE, Corporate Health Management, Ludwigshafen, Germany

Corresponding author: Dr. Julia Hiller, E-mail: julia.hiller@fau.de

Rationale for chosen exposure scenarios

The application scenarios (e.g. localization and size of exposure sites, PhE doses) were selected to ensure a reliable analyzation of penetrated PhE and its metabolites in urine and blood, while simultaneously keeping the exposure areas limited to one anatomic site and ensuring a safe exposure level. As previous studies on the metabolism and toxicokinetics of PhE (Eckert et al. 2024, 2025) indicated a low influence of the dose and route (oral or dermal) of PhE administration on its absorption, it was decided to keep the dermal loading dose per cm² skin fixed in order to ensure a uniform and comparable dispensation. A PhE level of 10.0 % in the ointment was chosen to allow a reduced exposure area of 200 cm² on the hands, while still ensuring reliable biomonitoring. Furthermore, the selected PhE exposure level (max. 1.4 mg/kg bw) falls safely below the derived no effect level (DNEL) for long-time dermal exposure in the general population (10.42 mg/kg bw; ECHA 2025).

Cited References:

- ECHA (European Chemicals Agency) (2025) 2-Phenoxyethanol. Last updated 03/07/25. https://echa.europa.eu/de/brief-profile/-/briefprofile/100.004.173, accessed on 05/07/25
- Eckert E, Jäger T, Hiller J Leibold E, Bader M, Göen T (2024) Biotransformation and toxicokinetics of 2-phenoxyethanol after oral exposure in humans: a volunteer study. Arch Toxicol 98:1771-1780. https://doi.org/10.1007/s00204-024-03717-2
- Eckert E, Jäger T, Leibold E, Bader M, Göen T, Hiller J (2025) Dermal penetration of 2-phenoxyethanol in humans: in vivo metabolism and toxicokinetics. Arch Toxicol 99(3):1095-1103. https://doi.org/10.1007/s00204-024-03938-5
