# Supplementary material for: Influence of the exposed anatomic sites on the human in vivo percutaneous absorption of the amphiphilic 2-phenoxyethanol
Source: Arch Toxicol. 2025 Oct 29;100(2):557–67. doi: 10.1007/s00204-025-04212-y (PMC12886243; doi:10.1007/s00204-025-04212-y)
Supplement: Supplementary file 2 — Supplementary file2 (PDF 392 KB) [file 204_2025_4212_MOESM2_ESM.pdf]

**Online Resource 2:**

Title: Influence of the exposed anatomic sites on the human in-vivo percutaneous absorption of the amphiphilic 2-phenoxyethanol

In: Archives of Toxicology

By: Julia Hiller[1]*, Elisabeth Eckert[1,2], Thomas Jäger[3], Michael Bader[3], Andrea Kaifie[1], Thomas Göen[1]

[1] Institute and Outpatient Clinic of Occupational, Social and Environmental Medicine, Friedrich-Alexander-Universität Erlangen-Nürnberg, Erlangen, Germany

[2] Bavarian Health and Food Safety Authority, Erlangen, Germany

[3] BASF SE, Corporate Health Management, Ludwigshafen, Germany

Corresponding author: Dr. Julia Hiller, E-mail: julia.hiller@fau.de

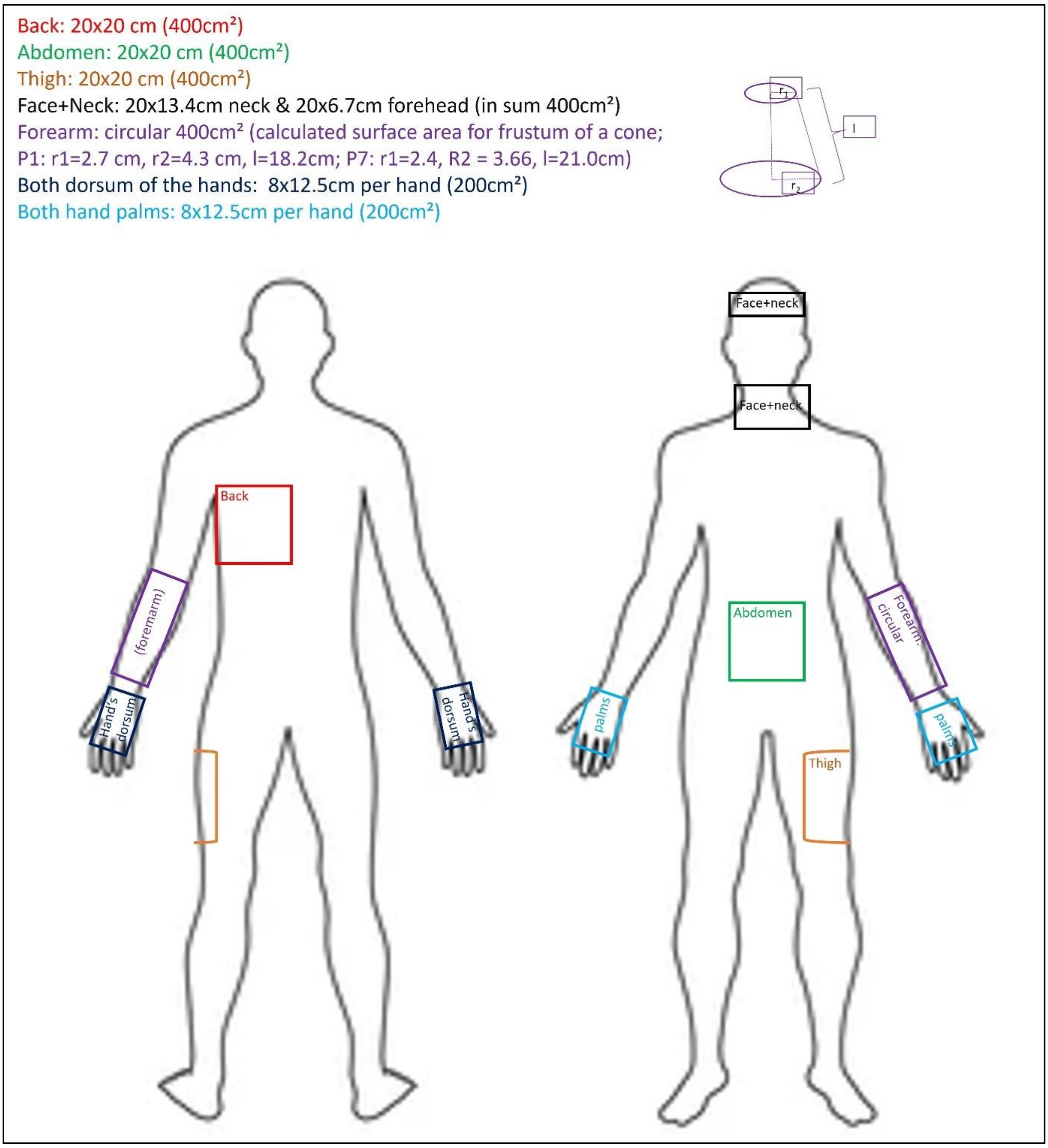


**Figure S1:** Illustration and area calculation specifics of compared exposure areas

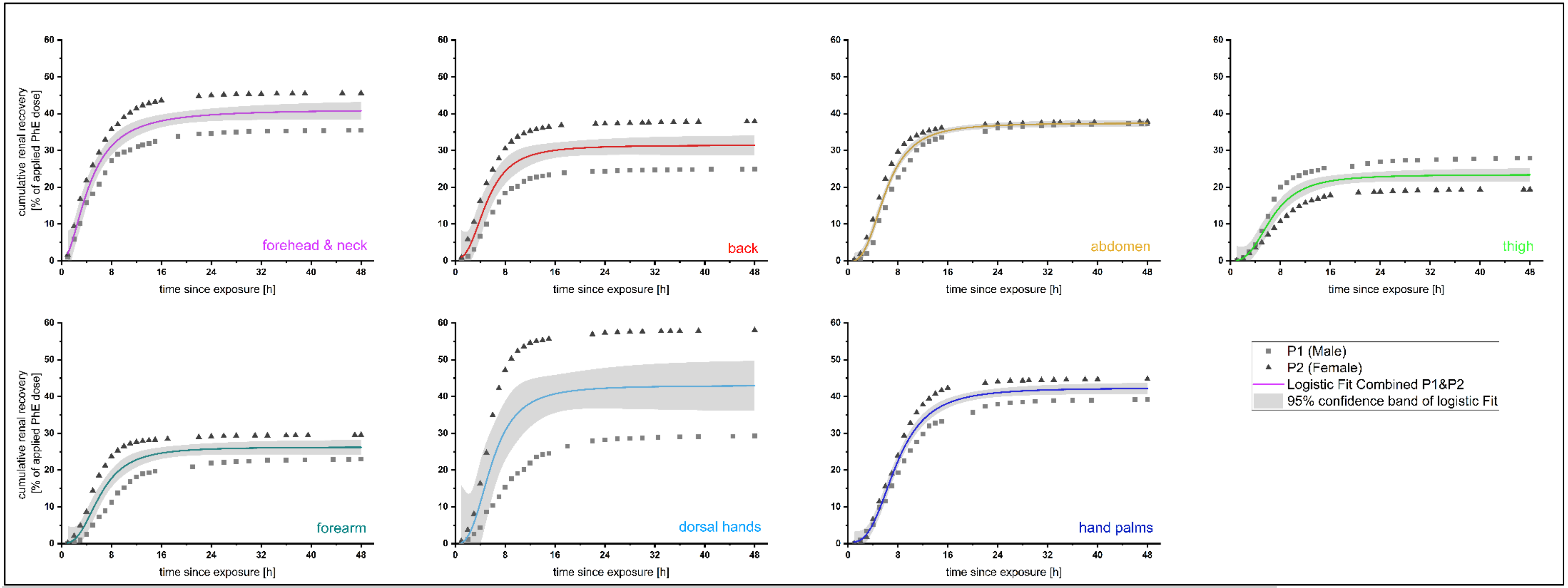


**Figure S2**: Cumulative renal recovery in % of applied dermal PhE dose per application site and individual volunteer data

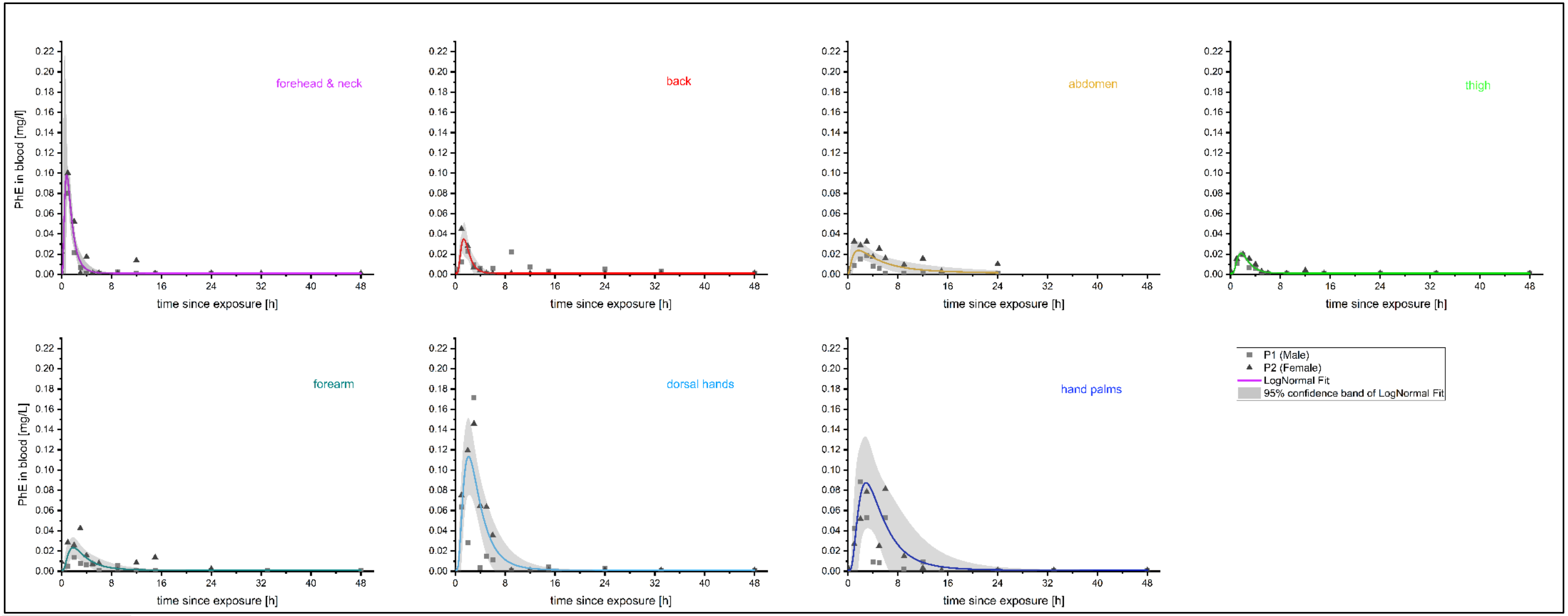


**Figure S3**: Site-specific elimination kinetics after dermal exposure with phenoxyethanol of PhE in blood; showing individual data point for both volunteers and a combined Lognormal fit.

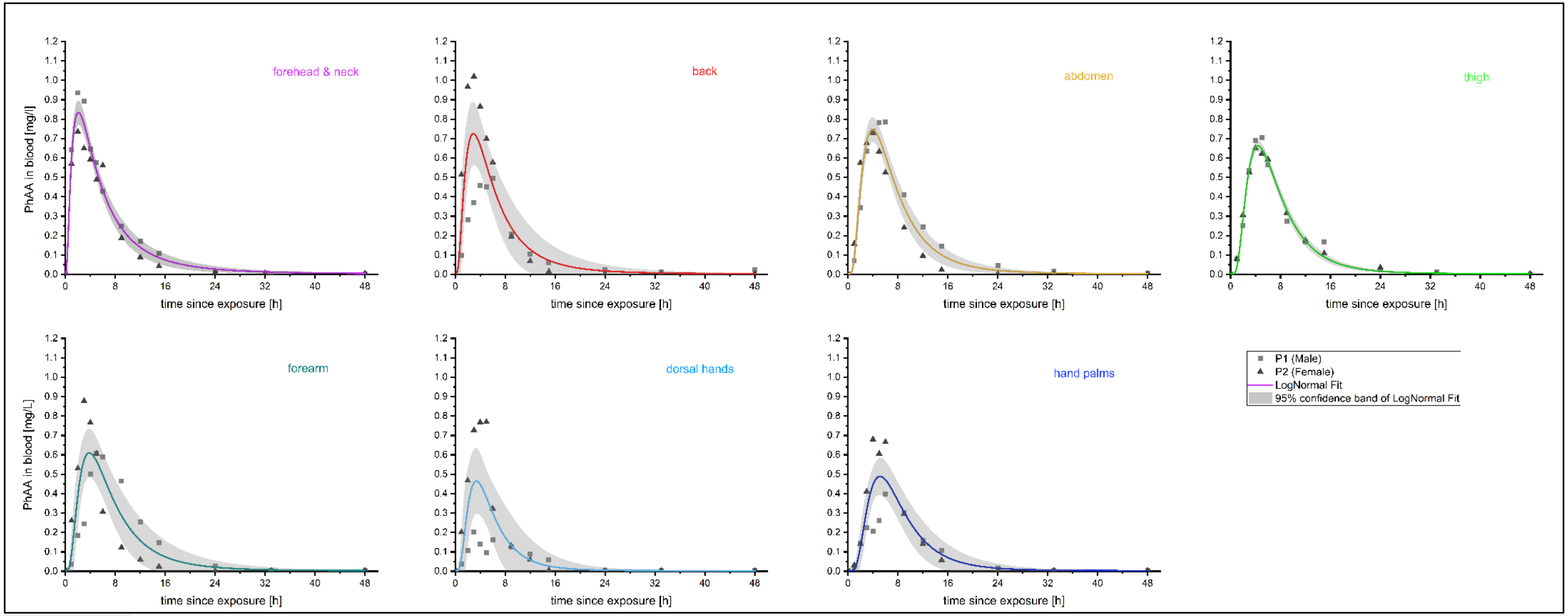


**Figure S4:** Site-specific elimination kinetics after dermal exposure with phenoxyethanol of PhAA in blood; showing individual data point for both volunteers and a combined Lognormal fit.

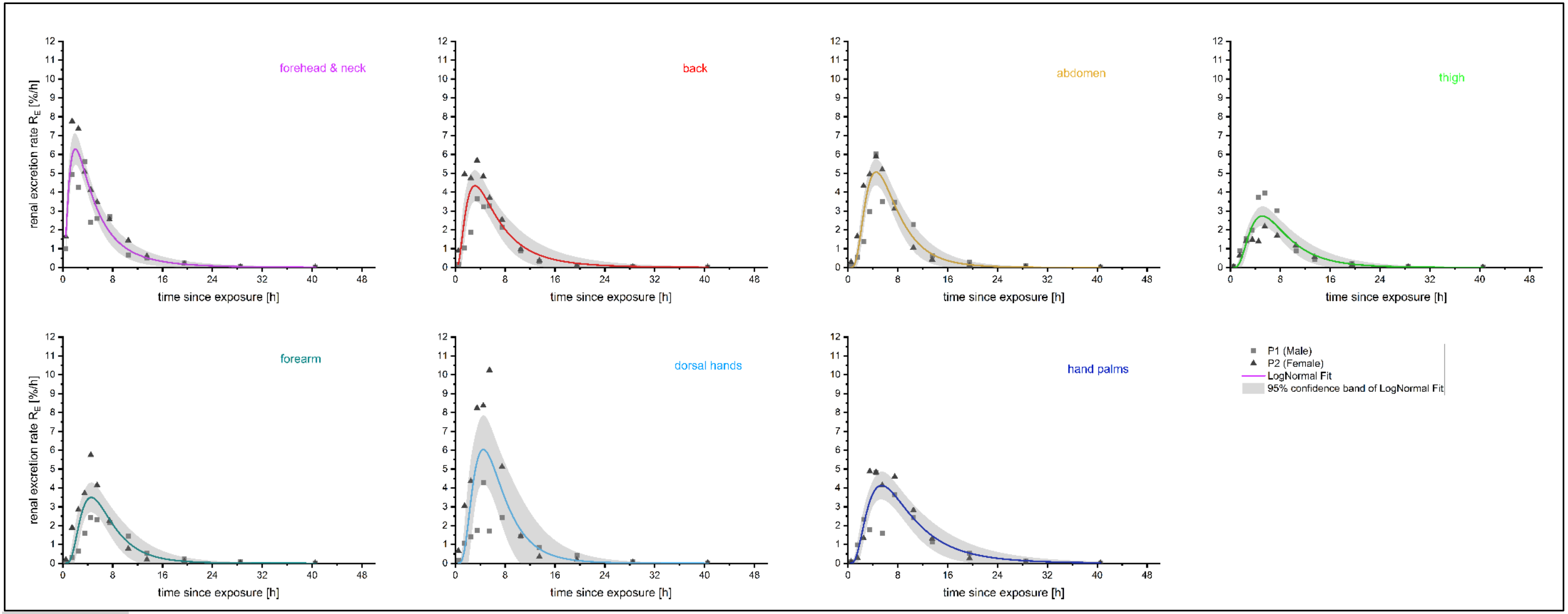


**Figure S5**: Site-specific elimination kinetics after dermal exposure with phenoxyethanol of the sum of its metabolites in urine; showing individual data point for both volunteers and a combined Lognormal fit
