# Supplementary material for: Influence of the exposed anatomic sites on the human in vivo percutaneous absorption of the amphiphilic 2-phenoxyethanol
Source: Arch Toxicol. 2025 Oct 29;100(2):557–67. doi: 10.1007/s00204-025-04212-y (PMC12886243; doi:10.1007/s00204-025-04212-y)
Supplement: Supplementary file 3 — Supplementary file3 (PDF 204 KB) [file 204_2025_4212_MOESM3_ESM.pdf]

Online Resource 3:

Title: Influence of the exposed anatomic sites on the human in-vivo percutaneous absorption of the amphiphilic 2-phenoxyethanol

In: Archives of Toxicology

By: Julia Hiller[1]*, Elisabeth Eckert[1,2], Thomas Jäger[3], Michael Bader[3], Andrea Kaifie[1], Thomas Göen[1]

[1] Institute and Outpatient Clinic of Occupational, Social and Environmental Medicine, Friedrich-Alexander-Universität Erlangen-Nürnberg, Erlangen, Germany

[2] Bavarian Health and Food Safety Authority, Erlangen, Germany

[3] BASF SE, Corporate Health Management, Ludwigshafen, Germany

Corresponding author: Dr. Julia Hiller, E-mail: julia.hiller@fau.de

Table S1: Subjects' characteristics and exposure doses (mean ± standard deviation)

Subject No.	Age [years], sex	Body weight [kg]	PhE dose per body weight for 400cm² sites	PhE dose per body weight for 200cm² sites
1	57, male	83	7.7 ± 0.2 µmol/kg bw (1.1 ± 0.02 mg/kg bw)	3.9 ± 0.01 µmol/kg bw (0.5 ± 0.00 mg/kg bw)
2	34, female	61	10.2 ± 0.5 µmol/kg bw (1.4 ± 0.07 mg/kg bw)	5.1 ± 0.05 µmol/kg bw (0.7 ± 0.01 mg/kg bw)

Table S2: Site-specific relative share of total renal recovered phenoxyethanol (PhE) metabolites at certain time points after a dermal PhE exposure and time after exposure until half of the total recovered amount was excreted. (SE = standard error)

	Relative share [%] of total recovered metabolites at									Time [h] until 50% of total excreted (t_{rec50}) ± SE
	2h	4h	6h	8h	12h	16h	24h	36h	48h	
**Neck & forehead**	17.1	45.0	64.6	76.5	88.2	93.3	97.3	99.3	100	4.45 ± 0.73
**Back**	9.6	36.0	61.7	77.5	91.1	95.8	98.7	99.7	100	5.08 ± 0.86
**Abdomen**	3.4	22.9	49.5	69.6	88.4	94.7	98.5	99.7	100	6.03 ± 0.22
**Thigh**	3.1	19.0	42.8	63.0	84.6	92.8	97.9	99.6	100	6.66 ± 0.72
**Forearm**	4.0	22.6	48.0	67.7	87.1	94.0	98.2	99.6	100	6.19 ± 0.70
**Dorsal hands**	4.6	25.7	52.4	71.5	88.9	94.9	98.5	99.7	100	5.81 ± 1.37
**Hand palms**	2.5	12.8	32.4	53.2	79.6	90.5	97.3	99.5	100	7.73 ± 0.32

Table S3: Peak levels of PhAA and PhE in blood and FUEsum in urine per anatomic site following dermal PhE administration to two volunteers

**Anatomic site**	**C_{max} [mg/L] / FUEsum [%/h]**		
	**PhE**	**PhAA**	**∑PhAA+ 4OH-PhAA**
Neck & forehead	0.098	0.83	6.28
Back	0.035	0.73	4.34
Abdomen	0.024	0.75	5.06
Thigh	0.022	0.66	2.73
Forearm	0.023	0.60	3.49
Dorsal hands	0.113	0.47	6.03
Hand palms	0.087	0.49	4.12

Table S4: Half-lives of PhAA in blood and FUEsum in urine per anatomic site following dermal PhE administration to two volunteers (SE = Standard error range)

**Anatomic site**	**$t_{1/2}$ [h]**	
	**PhAA in blood**	**∑PhAA+ 4OH-PhAA in urine**
Neck & forehead	3.43 (SE: 3.17 - 3.73)	4.41 (SE: 4.21 - 4.64)
Back	3.03 (SE: 2.61 - 3.62)	4.39 (SE: 4.08 - 4.76)
Abdomen	3.08 (SE: 2.57 - 3.85)	4.49 (SE: 4.13 - 4.92)
Thigh	4.37 (SE: 4.12 - 4.64)	4.67 (SE: 4.38 - 5.01)
Forearm	3.22 (SE: 2.66 - 4.08)	4.49 (SE: 4.13 - 4.93)
Dorsal hands	2.79 (SE: 2.46 - 3.22)	4.44 (SE: 4.07 - 4.88)
Hand palms	2.89 (SE: 2.61 - 3.22)	4.37 (SE: 4.11 - 4.66)

Table S5: Compilation of literature data on percutaneous absorption measured by total recovery in urine and related to a reference site (forearm or upper outer arm) for a selection of different anatomic sites (excluding genitalia, axilla, ear canal, fossa cubitalis) (adapted from *Bormann & Maibach 2020*)

**Substances**	**2-phenoxy-ethanol**	**Hydro-cortisone**	**Carba-ryl**	**Para-thion**	**Mala-thion**	**1-OH-pyrene**	**Benzoic acid**	**Benzoic acid**	**Benzoic acid**	**Benzoic acid sodium salt**	**Caffeine**	**Acetyl-salicylic acid**
**Reference**	*this study*	*Feldmann & Maibach 1967*	*Maibach et al. 1971*	*Maibach et al. 1971*	*Maibach et al. 1971*	*VanRooij et al. 1993*	*Rougier et al. 1988, cited by Bormann et al. 2020*	*Rougier et al. 1986*	*Lotte et al. 1987*	*Lotte et al. 1987*	*Lotte et al. 1987*	*Lotte et al. 1987*
	Referred to forearm							*Referred to outer upper arm*				
**Postauricular**				3.9			4.1		2.5	2.5	1.0	1.7
**Scalp**		3.5		3.7								
**Face (forehead) & neck**	1.6											
**Forehead**		6		4.2	3.4		4.9	2.9	2.9	3.1	1.8	2.1
**Jaw angle**		13	1.0	3.9								
**Neck**						1.3						
**Back**	1.2	1.7						0.9				
**Abdomen (belly)**	1.4			2.1	1.4	1.1	2.3	1.6	1.6	1.9	0.6	1.0
**Chest/ shoulder**								1.2				
**Upper outer arm**							1.7	1.0	1.0	1.0	1.0	1.0
**Forearm (circular / not specified)**	1.0		1.0	1.0	1.0	1.0						

**Substances**	**2-phenoxy-ethanol**	**Hydro-cortisone**	**Carba-ryl**	**Para-thion**	**Mala-thion**	**1-OH-pyrene**	**Benzoic acid**	**Benzoic acid**	**Benzoic acid**	**Benzoic acid sodium salt**	**Caffeine**	**Acetyl-salicylic acid**
**Reference**	*this study*	*Feldmann & Maibach 1967*	*Maibach et al. 1971*	*Maibach et al. 1971*	*Maibach et al. 1971*	*VanRooij et al. 1993*	*Rougier et al. 1988, cited by Bormann et al. 2020*	*Rougier et al. 1986*	*Lotte et al. 1987*	*Lotte et al. 1987*	*Lotte et al. 1987*	*Lotte et al. 1987*
	Referred to forearm							*Referred to outer upper arm*				
**Forearm (ventral)**		1.0					1.0 (mid arm); 0.6 (at elbow), 2.2 (at wrist)					
**Forearm (dorsal)**		1.1										
**Hand dorsum**	1.6											
**Hand palm**	1.6	0.8		1.3	0.9							
**Hand dorsum/ ventral**				2.4	1.8	0.8						
**Thigh**	0.9							1.3				
**Calf**						1.2						
**Ankle (lateral or medial)**		0.4										
**Foot arch (plantar)**		0.1		1.6	1.0							

Cited References:

- Feldmann RJ, Maibach HI (1967) Regional variation in percutaneous penetration of 14C cortisol in man. J Invest Dermatol 48(2):181-3. https://doi.org/10.1038/jid.1967.29

- Lotte C, Rougier A, Wilson DR, Maibach HI (1987) In vivo relationship between transepidermal water loss and percutaneous penetration of some organic compounds in man: effect of anatomic site. Arch Dermatol Res 279(5):351-6. https://doi.org/10.1007/BF00431230
- Maibach HI, Feldman RJ, Milby TH, Serat WF (1971) Regional variation in percutaneous penetration in man. Pesticides. Arch Environ Health 23(3):208-11. https://doi.org/10.1080/00039896.1971.10665987
- Rougier A, Dupuis D, Lotte C, Roguet R, Wester RC, Maibach HI (1986) Regional variation in percutaneous absorption in man: measurement by the stripping method. Arch Dermatol Res 278(6):465-9. https://doi.org/10.1007/BF00455165
- Rougier A, Lotte C, Corcuff P, Maibach HI (1988) Relationship between skin permeability and corneocyte size according to anatomic site, age, and sex in man. J Soc Cosmet Chem 39:15-26.
    - Cited by: Bormann JL, Maibach HI (2020) Effects of anatomical location on in vivo percutaneous penetration in man. Cutan Ocul Toxicol 39(3):213-222. https://doi.org/10.1080/15569527.2020.1787434
- VanRooij JG, De Roos JH, Bodelier-Bade MM, Jongeneelen FJ (1993) Absorption of polycyclic aromatic hydrocarbons through human skin: differences between anatomical sites and individuals. J Toxicol Environ Health 38(4):355-68. https://doi.org/10.1080/15287399309531724
